# Case Report: Compound heterozygous mutations in the *IDUA* gene causing mucopolysaccharidosis type I with uterine developmental abnormality

**DOI:** 10.3389/fped.2026.1768349

**Published:** 2026-02-27

**Authors:** Yuwan Xu, Jing Li, Liuxi Wang, Sancong Pan, Yajie Fan

**Affiliations:** 1 Changzhi Medical College, Changzhi, China; 2 Department of Endocrinology, Jincheng People’s Hospital, Jincheng, China; 3 Department of Nephrology, Jincheng People’s Hospital, Jincheng, China; 4 Department of Cardiology, Jincheng People’s Hospital, Jincheng, China

**Keywords:** case report, compound heterozygous mutation, *IDUA* gene, mucopolysaccharidosis type I, short stature, uterine developmental abnormality

## Abstract

Mucopolysaccharidosis (MPS) represents a group of rare inherited metabolic disorders characterized by abnormal accumulation of glycosaminoglycans (GAGs) due to deficiencies of lysosomal enzymes. Mucopolysaccharidosis type I (MPS I) is caused by biallelic pathogenic variants in the *IDUA* gene and is inherited in an autosomal recessive pattern. The *IDUA* gene is located on chromosome 4p16.3 and encodes the lysosomal enzyme *α*-L-iduronidase, which plays a critical role in the degradation of GAGs, particularly dermatan sulfate and heparan sulfate. Reduced or absent *IDUA* enzymatic activity leads to the progressive accumulation of undegraded substrates within lysosomes, resulting in multisystem organ involvement. Based on clinical severity, MPS I is traditionally classified into three phenotypic subtypes: the severe form (Hurler syndrome), the intermediate form (Hurler–Scheie syndrome), and the attenuated form (Scheie syndrome, MPS I-S). This report describes a 13-year-old female patient in whom compound heterozygous pathogenic variants in the *IDUA* gene were identified by genetic testing, and whose clinical manifestations were consistent with the MPS I-S. In addition to typical skeletal and joint abnormalities, the patient also presented with uterine developmental abnormality. Currently, there is no definitive evidence supporting a direct causal relationship between MPS I and uterine developmental abnormalities; however, this case suggests a potential association between MPS I and reproductive system developmental abnormalities. This case may help further expand the phenotypic spectrum of MPS I and enhance clinical awareness of its multisystem involvement.

## Introduction

Mucopolysaccharidosis (MPS) represents a group of rare lysosomal storage disorders that are classified into types I, II, III, IV, VI, VII, and IX according to the specific lysosomal enzyme deficiency involved. Among these, mucopolysaccharidosis type I (MPS I) is caused by pathogenic variants in the gene encoding α-L-iduronidase (*IDUA*). The *IDUA* gene is located on chromosome 4p16.3 and comprises 14 exons and 13 introns, encoding a precursor protein consisting of 653 amino acids. MPS I is inherited in an autosomal recessive manner and requires biallelic pathogenic variants for disease manifestation. *IDUA* is a key lysosomal hydrolase involved in the degradation of glycosaminoglycans (GAGs), primarily responsible for removing terminal α-L-iduronic acid residues from dermatan sulfate and heparan sulfate. When *IDUA* activity is deficient, incompletely degraded GAGs progressively accumulate within lysosomes and the extracellular matrix, leading to cellular dysfunction and multisystem clinical manifestations, including coarse facial features, corneal clouding, upper airway obstruction, hepatosplenomegaly, skeletal deformities, and cardiovascular involvement (1). The estimated incidence of MPS I is approximately 1 per 100,000 live births (2). More than 200 pathogenic variants of the *IDUA* gene have been reported to date, contributing to the marked clinical heterogeneity of the disease. Based on disease severity, MPS I is traditionally classified into Hurler syndrome, Hurler–Scheie syndrome, and Scheie syndrome (MPS I-S, attenuated form) (3). Among these, MPS I-S typically presents with later onset, slower disease progression, and relatively preserved cognitive function, although multiple organ systems may still be affected. Previous studies have primarily focused on the involvement of the skeletal, cardiovascular, and nervous systems in MPS I, whereas abnormalities in reproductive system development have rarely been reported. In this study, we report a case of attenuated MPS I caused by compound heterozygous variants in the *IDUA* gene, accompanied by uterine developmental abnormality. This case aims to further expand the phenotypic spectrum of MPS I and enhance clinical awareness of its multisystem manifestations.

## Case description

The patient is a 13-year-old female, the first child born to healthy, unrelated parents with no family history of MPS I. Her birth length is unknown, and she weighed 3.3 kg at birth. She was breastfed until 1 year and 1 month of age, teethed at 4 months, began speaking at 6 months, and started walking at 1 year. She has been shorter than her peers since infancy. At 4.5 years of age, the child developed inability to extend fingers in both hands. At age 5, bilateral genu varum deformity appeared. At age 6, the child was examined at another hospital for the aforementioned symptoms. LSD enzyme analysis revealed significantly reduced α-L-iduronidase activity at 0.3 nmol/(g·min). Galactosamine-6-sulfatase activity was 18.1 nmol/(mg·h), showing mildly low levels. Combined with the enzyme assay results, this suggests a lysosomal storage disorder due to α-L-iduronidase deficiency, with a high likelihood of MPS. No specific intervention or treatment was administered at that time. At age 8.5 years, restricted extension of both upper limbs developed. At age 11, a school physical examination revealed hyperopia, which has been corrected with glasses.

At admission, physical examination revealed the patient's height was 126 cm and weight as 26 kg. She exhibited widened interpupillary distance and positive epicanthal folds. Multiple pigmented nevi are visible on her face and neck. No Mongolian spots are present on the skin of her lumbosacral region and buttocks. She has a small jaw, widened intercostal spaces, cubitus valgus, limited abduction of both upper limbs, restricted flexion and extension of the finger joints in both hands presenting as “claw hands,” and bilateral genu varum deformity. Breasts: Tanner stage I. Pubic hair: Tanner stage I. Physical examination of the heart, lungs, and abdomen revealed no significant abnormalities. Neurological examinations showed no positive findings.

Cortisol, total type I collagen N-terminal propeptide, and adrenocorticotropic hormone (ACTH) levels were all within normal ranges. 25-hydroxyvitamin D was 11.70 ng/mL, and insulin-like growth factor 1 (IGF-1) was 128 ng/mL. Luteinizing hormone 0.39 mIU/mL, follicle-stimulating hormone 1.31 mIU/mL, estradiol <5.000 pg/mL, parathyroid hormone 48.4 pg/mL, growth hormone 0.511 ng/mL, peripheral blood chromosome karyotype analysis 46, XX.

**Figure 1:**
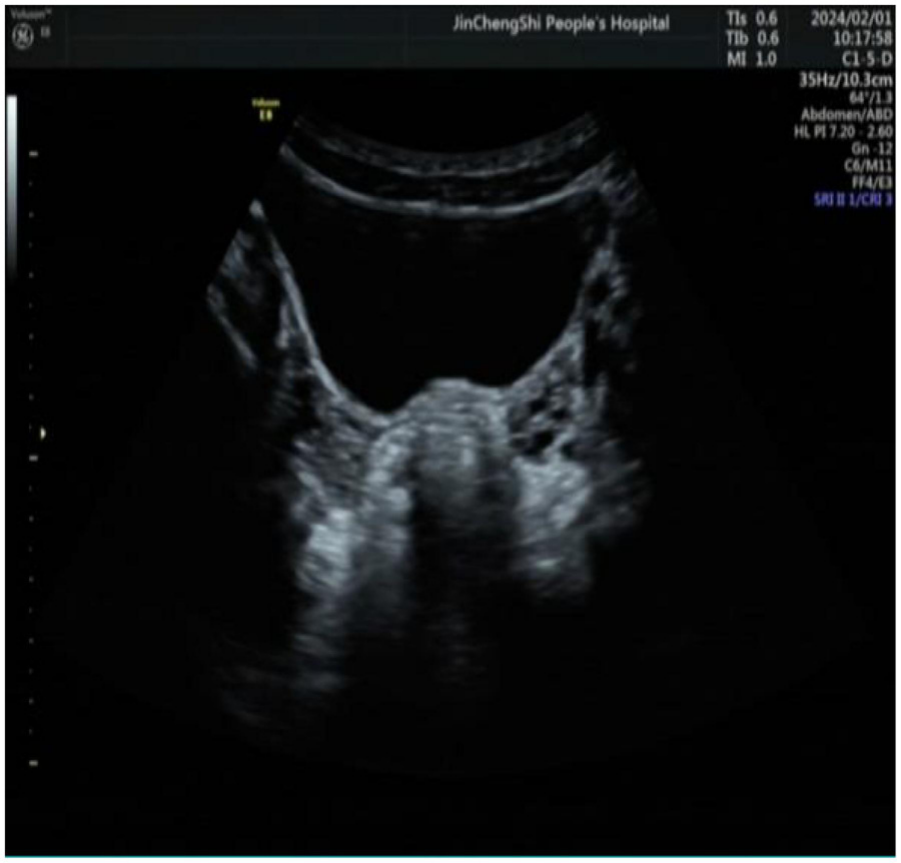
Ultrasound of the patient's uterus and bilateral adnexa.

Echocardiography revealed mitral valve prolapse of the anterior leaflet, increased anterior mitral flow velocity, and mild regurgitation. Transabdominal ultrasound of the uterus and bilateral adnexa showed no uterine structure visible. Bilateral ovaries were visualized with dimensions approximately: left 3.2 cm × 1.7 cm × 2.4 cm, right 3.1 cm × 1.5 cm × 1.7 cm. Color Doppler flow imaging (CDFI) revealed no abnormal blood flow signals (Figure 1).

Thyroid ultrasound revealed a left lobe thickness of approximately 0.8 cm, a right lobe thickness of approximately 0.8 cm, and an isthmus thickness of approximately 0.2 cm. The thyroid parenchyma exhibited uniform echogenicity with no significant masses visible. CDFI indicated normal blood flow distribution within the thyroid, and no markedly enlarged lymph nodes were observed in the drainage areas.

**Figure 2:**
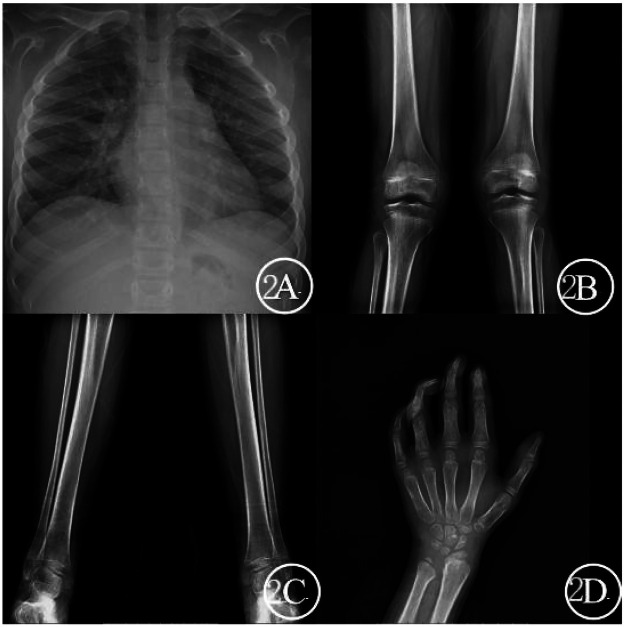
Pediatric patient radiographic examination **(A)** chest x-ray, anterior-posterior view; **(B,C)** full-length x-rays of both lower limbs, anterior-posterior view; **(D)** left hand x-ray, anterior-posterior view.

Anterior-posterior chest radiograph reveals a “paddle-shaped” sternum, with no significant abnormalities in thoracic vertebral bodies (Figure 2A). Full-length anteroposterior radiographs of both lower limbs show bilateral genu varum deformity (Figures 2B,C). Anteroposterior radiograph of the left hand shows bone age consistently with approximately 8 years; the radial epiphysis exhibits incomplete morphology (Figure 2D). Pituitary MRI reveals no significant abnormalities in plain scanning, with adenoid hypertrophy.

**Figure 3:**
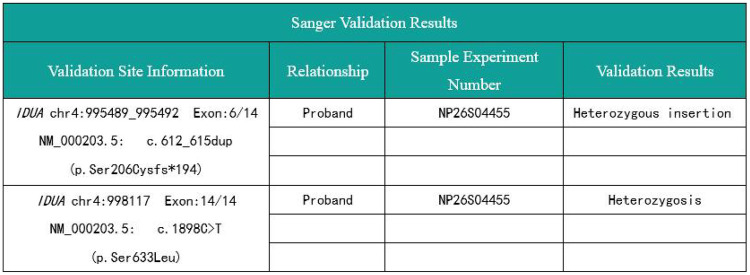
Sanger sequence read of patient.

**Figure 4:**
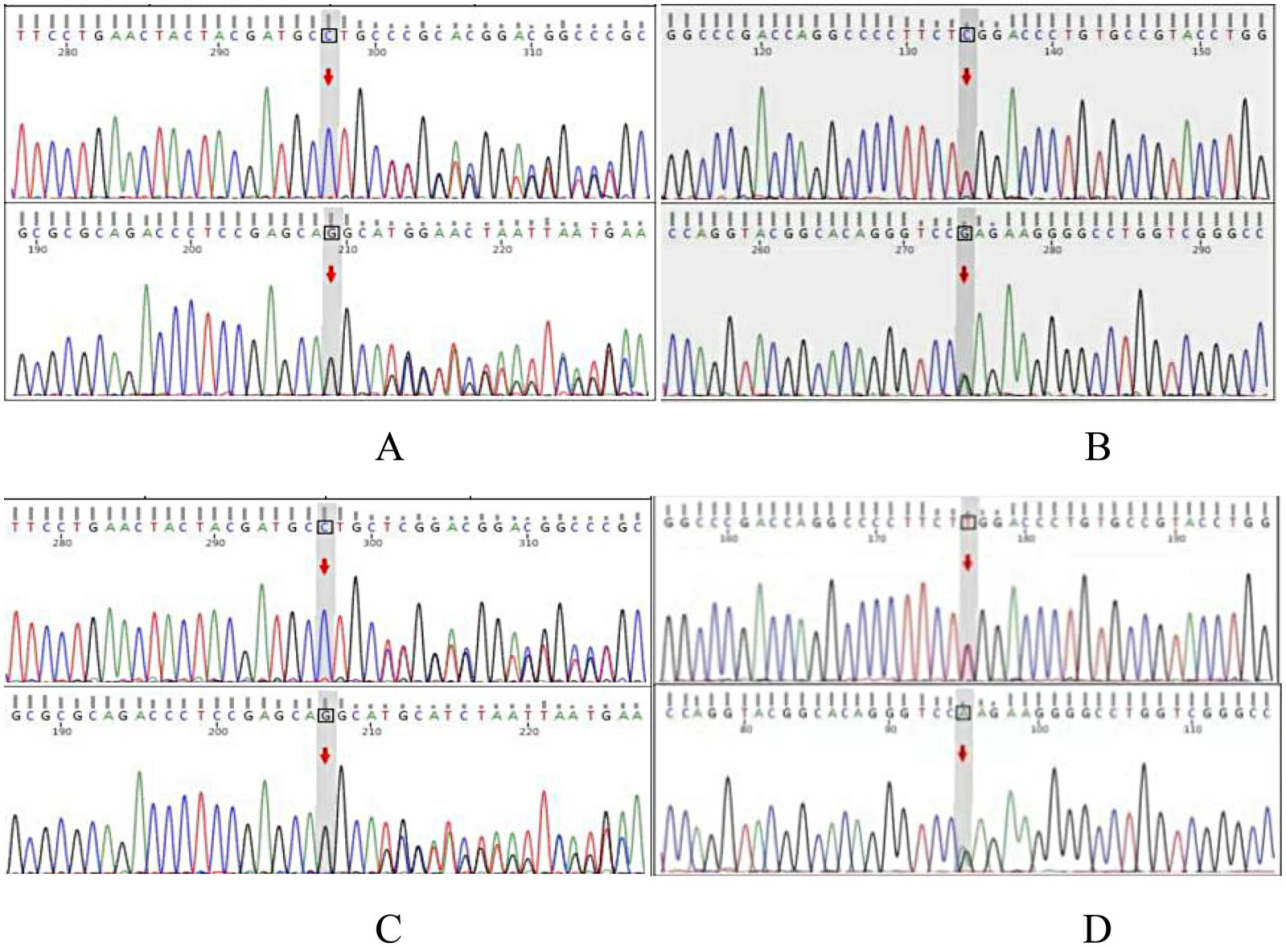
*IDUA* gene sequencing analysis of the patient and parents **(A)** patient c.612_615dup heterozygous insertion variant; **(B)** patient c.1898C>T heterozygous missense variant; **(C)** patient's father c.612_615dup heterozygous insertion variant; **(D)** patient's mother c.1898C>T heterozygous variant.

Peripheral venous blood was collected from the patient for whole-exome sequencing of inherited disorders. To confirm the variant phase, Sanger sequencing was performed at the specific variant locus in the parents of the patient, who exhibited no clinical phenotype. Whole-exome sequencing results (Figure 3) revealed a c.612_615dup (p.Ser206Cysfs*194) variation in the patient's *IDUA* gene, representing an insertion-induced frameshift mutation within the coding region (Figure 4A). This mutation causes a repeat or insertion of nucleotides at positions 612 to 615, resulting in a frameshift in the protein starting at amino acid 206 (Ser). This frameshift leads to a premature termination codon at position 194 following the shift. This results in truncated proteins or activating nonsense-mediated mRNA decay (NMD), leading to significantly reduced or complete loss of *IDUA* enzyme function. If this abnormal mRNA escapes the NMD pathway, it may still be translated into a functionally abnormal truncated protein, further exacerbating enzyme activity impairment.

Sanger sequencing validation confirmed that this variant is in a heterozygous state, with the father carrying a heterozygous variant at this locus (Figure 4B) and no such mutation detected in the mother, which is consistent with an autosomal recessive inheritance pattern. This variant is located in a key functional segment of the coding region of the *IDUA* gene, and there are currently few relevant reports in the literature or population databases such as gnomAD. According to the Standards and Guidelines for the Interpretation of Sequence Variants issued by the American College of Medical Genetics and Genomics (ACMG) (4), the *IDUA* gene c.612_615dup variant is classified as PVS1 (Pathogenic Very Strong) and PM2 (Moderate Pathogenicity) and is defined as a likely pathogenic variant based on the available evidence.

The patient also harbors a missense variant in the *IDUA* gene: c.1898C>T (p. Ser633Leu) (Figure 4C). Sanger sequencing validation (Figure 3) showed that this mutation is located in the coding region of the *IDUA* gene on chromosome 4 (chr4:998117), resulting from a C→T nucleotide substitution. This substitution leads to the replacement of serine (Ser) with leucine (Leu) at amino acid position 633 of the encoded protein, which may potentially affect protein structure and function. The patient's mother is a heterozygous carrier of this variant (Figure 4D), while the father does not carry it. In accordance with the ACMG guidelines (4), the *IDUA* gene c.1898C>T variant is classified as PS1 (Strong Pathogenicity). Additionally, this variant has been reported in 3 heterozygous individuals in the large-scale population frequency database gnomAD, with no homozygous individuals identified, indicating its rarity in the general population and further supporting its pathogenicity. Based on the comprehensive clinical and genetic evidence, this variant is classified as a pathogenic variant.

## Diagnosis

The patient was an adolescent female who had exhibited short stature since early childhood and gradually developed ocular and joint involvement as the disease progressed. Physical examination revealed mildly dysmorphic facial features, including hypertelorism, epicanthal folds, and micrognathia. Multiple pigmented nevi were observed on the face and neck, while no Mongolian spots were noted over the lumbosacral or gluteal regions. Additional findings included widened intercostal spaces, cubitus valgus, limited abduction of both upper limbs, and restricted flexion and extension of the finger joints with a claw-hand deformity. Secondary sexual characteristics were underdeveloped, with both breast and pubic hair development corresponding to Tanner stage I. Imaging studies demonstrated a paddle-shaped sternum on chest radiography. Long-bone radiographs of the lower extremities revealed bilateral genu valgum deformities. Bone age assessment of the left hand indicated a bone age of approximately 8 years, which was markedly delayed relative to chronological age. Laboratory evaluation demonstrated markedly reduced α-L-iduronidase activity on lysosomal enzyme analysis, accompanied by mildly decreased galactosamine-6-sulfatase activity. Subsequent whole-exome sequencing identified two heterozygous pathogenic variants in the *IDUA* gene, including a frameshift variant (c.612_615dup) and a missense variant (c.1898C>T). Based on the patient's clinical manifestations, enzymatic findings, and genetic testing results, a definitive diagnosis of mucopolysaccharidosis type I was established.

## Treatment

Vitamin D Deficiency: administer vitamin D2 soft capsules at 800 IU daily (oral intake). Regularly recheck serum 25-hydroxyvitamin D levels, with a target range of 30–50 ng/mL.

Hearing: audiometric testing revealed mild sensorineural hearing loss, which does not impact daily life. Schedule regular follow-up visits with the otolaryngology department.

Ophthalmology: conduct routine ophthalmic follow-up examinations, including funduscopy.

Cardiology: the patient's cardiac ejection fraction is within the normal range and does not currently affect daily activities. Perform regular re-evaluations with echocardiography.

Gynecology: monitor sex hormone levels and conduct imaging studies of the uterus and adnexa. Initiate exogenous estrogen intervention if clinically indicated.

Skeletal Abnormalities: implement physical therapy and rehabilitation training.

## Treatment outcomes, follow-up, and prognosis

### Follow-up 15 months after discharge

Reproductive hormones: Luteinizing hormone (LH) 2.54 mIU/mL, follicle-stimulating hormone (FSH) 4.24 mIU/mL, estradiol (E2) 47.50 pg/mL, progesterone < 0.050 ng/mL.

**Figure 5:**
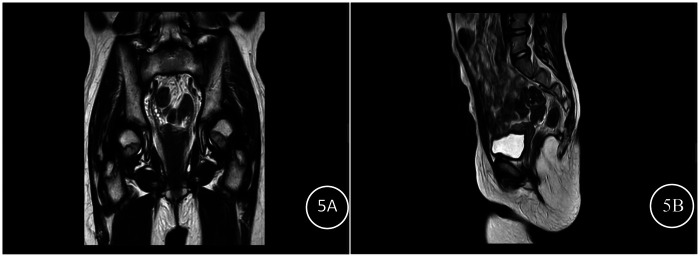
Pediatric MRI findings **(A)** pelvic MRI coronary view; **(B)** pelvic MRI T2 sagittal view.

Pelvic magnetic resonance imaging (MRI): A small uterine shadow was visualized superior to the urinary bladder, with dimensions of approximately 1.6 cm × 2.6 cm × 1.9 cm (left-right diameter × anteroposterior diameter × craniocaudal diameter) (Figure 5A). A linear T2-weighted imaging (T2WI) hyperintense signal was observed within the uterus (Figure 5B). Bilateral ovaries were clearly visualized without abnormal signals; no other significant abnormalities were detected. Imaging conclusion: Small uterine volume, consistent with uterine developmental abnormality, most likely infantile uterus.

## Outcomes and prognosis

Vitamin D supplementation failed to improve the patient's skeletal deformities, and physical therapy/rehabilitation training yielded limited efficacy. Regarding hearing and cardiac function, surgical intervention by otolaryngologists or cardiac surgeons may be required as the progression of MPS exacerbates.

## Discussion

MPS I is a lysosomal storage disorder caused by deficiency of *IDUA*, leading to the progressive accumulation of GAGs within lysosomes and the extracellular matrix, ultimately resulting in multisystem involvement. Previous studies have demonstrated a close association between the type of *IDUA* gene variants and disease severity (3).Genetic testing in this patient identified compound heterozygous variants in the *IDUA* gene, including a truncating frameshift variant (c.612_615dup, p.Ser206Cysfs*194) and a missense variant (c.1898C>T, p.Ser633Leu), inherited from each parent, consistent with an autosomal recessive inheritance pattern. Considering the patient's preserved cognitive function, slow disease progression, and relatively mild clinical manifestations, the phenotype is most consistent with MPS I-S.

MPS I is traditionally classified into Hurler syndrome (severe form), Hurler–Scheie syndrome (intermediate form), and Scheie syndrome (attenuated form). The attenuated form of MPS I typically presents with later onset, slower disease progression, and relatively preserved cognitive function; however, skeletal deformities, joint stiffness, and cardiac involvement may still occur (5). In the present case, the patient exhibited no obvious dysmorphic facial features, with clinical manifestations mainly consisting of skeletal abnormalities and mild cardiac involvement, and no evidence of significant airway involvement or recurrent infections, further supporting the diagnosis of MPS I-S phenotype. Additionally, the patient demonstrated mildly reduced galactosamine-6-sulfatase activity. Previous studies have indicated that mild reductions in lysosomal enzyme activity may be observed in heterozygous carriers or in pseudodeficiency states, in which *in vitro* enzymatic activity may fall below the normal reference range but typically does not result in clinical disease (3).Therefore, we consider that the mildly reduced enzyme activity is more likely attributable to carrier status or methodological variability in testing, rather than representing a clinically significant enzymatic deficiency. During follow-up, uterine developmental abnormality was identified in the patient. However, the initial ultrasound examination failed to visualize the uterus, whereas subsequent pelvic magnetic resonance imaging demonstrated an infantile uterus rather than true uterine agenesis. This discrepancy in imaging findings suggests that the uterine developmental abnormality may be associated with delayed pubertal development or growth retardation related to chronic disease. Currently, there is no direct evidence indicating that MPS I causes uterine developmental abnormalities; therefore, in this study, this finding is considered a potentially associated but not causally established clinical observation. Based on the pathophysiological characteristics of MPS, several hypothetical mechanisms may be proposed. First, glycosaminoglycan deposition within the central nervous system may affect hypothalamic–pituitary–gonadal axis function, thereby interfering with gonadotropin secretion and pubertal development (6, 7). Second, chronic disease status and long-term metabolic burden may alter physiological resource allocation, potentially delaying reproductive system development. In addition, glycosaminoglycan accumulation may affect smooth muscle and stromal tissue architecture (8); however, histopathological evidence from uterine tissue is currently lacking to support this hypothesis.Alternatively, the patient may have constitutional delay of puberty, which is typically associated with genetic factors and individual developmental variability, and in most cases, normal pubertal development is eventually achieved. To further elucidate the underlying mechanisms, histopathological evaluation of uterine and pituitary tissues could theoretically be considered. However, given the limited clinical diagnostic and therapeutic value of biopsy in this patient, and the guardian's refusal of the procedure, invasive investigations were not pursued based on ethical considerations and individualized patient management. At present, a strategy of regular follow-up has been adopted, including dynamic monitoring of sex hormone levels and serial pelvic imaging to assess uterine development. Continued longitudinal observation is planned to further explore the potential underlying mechanisms. Regarding neurocognitive function, the Montreal Cognitive Assessment (MoCA) was performed in this patient. The patient achieved a total score of 27, which is above the commonly accepted cutoff value for normal cognitive function (≥26), indicating no evident neurocognitive impairment at present. However, given the progressive nature of MPS, long-term follow-up is required to monitor potential changes in cognitive and behavioral function. The patient has experienced persistent short stature, which may be related to skeletal structural abnormalities, endocrine dysfunction, and the chronic disease state. Previous studies have reported that growth hormone deficiency and pubertal abnormalities may occur in patients with MPS I; however, the effectiveness of growth hormone therapy remains uncertain in the absence of confirmed growth hormone deficiency (6, 9).

Currently, the main treatment strategies for MPS I include enzyme replacement therapy (ERT) and hematopoietic stem cell transplantation (HSCT). ERT can improve non-neurological clinical manifestations and delay disease progression; however, due to its limited ability to effectively cross the blood–brain barrier, its therapeutic effect on central nervous system involvement remains limited (6, 10). HSCT can improve cognitive function and multisystem manifestations when performed in the early stages of the disease, but its clinical indications are primarily restricted to patients with severe MPS I (11). The patient in this case exhibited relatively mild clinical manifestations, consistent with the MPS I-S phenotype. After comprehensive evaluation, ERT was recommended to improve non-neurological manifestations. However, the patient's guardians declined ERT because of concerns regarding financial burden and long-term treatment safety. Therefore, regular follow-up was adopted, with individualized symptomatic management implemented according to disease progression. In summary, mucopolysaccharidosis represents a group of rare inherited metabolic disorders characterized by multisystem involvement, progressive disease course, and generally unfavorable prognosis. Early recognition, prompt diagnosis, and standardized treatment, combined with regular follow-up monitoring, are essential for delaying disease progression and improving patients' quality of life. For high-risk individuals with a family history of MPS, genetic counseling and molecular genetic testing are of significant importance for disease prevention.

## Conclusion

This case suggests that, in addition to typical multisystem involvement, patients with MPS I-S may have a potential risk of reproductive system developmental abnormalities. However, definitive causal evidence is currently lacking, and this finding should be interpreted with caution. This case further expands the phenotypic spectrum of MPS I and highlights the clinical importance of early diagnosis, long-term follow-up, and multidisciplinary management in improving patient outcomes.

## Data Availability

The datasets presented in this study can be found in online repositories. The names of the repository/repositories and accession number(s) can be found in the article/Supplementary Material.
